# Use of Terrestrial Laser Scanner for Rigid Airport Pavement Management

**DOI:** 10.3390/s18010044

**Published:** 2017-12-26

**Authors:** Maurizio Barbarella, Fabrizio D’Amico, Maria Rosaria De Blasiis, Alessandro Di Benedetto, Margherita Fiani

**Affiliations:** 1 Department of Civil, Chemical, Environmental, and Materials Engineering—Advanced Research Center on Electronic Systems, University of Bologna, 40136 Bologna, Italy; 2 Department of Engineering, University of Roma TRE, 00146 Rome, Italy; fabrizio.damico@uniroma3.it (F.D.); mariarosaria.deblasiis@uniroma3.it (M.R.D.B.); alessandro.dibenedetto@uniroma3.it (A.D.B.); 3 Department of Civil Engineering, University of Salerno, 84084 Fisciano (SA), Italy; m.fiani@unisa.it

**Keywords:** terrestrial laser scanner, concrete pavement, faulting, algorithms, software

## Abstract

The evaluation of the structural efficiency of airport infrastructures is a complex task. Faulting is one of the most important indicators of rigid pavement performance. The aim of our study is to provide a new method for faulting detection and computation on jointed concrete pavements. Nowadays, the assessment of faulting is performed with the use of laborious and time-consuming measurements that strongly hinder aircraft traffic. We proposed a field procedure for Terrestrial Laser Scanner data acquisition and a computation flow chart in order to identify and quantify the fault size at each joint of apron slabs. The total point cloud has been used to compute the least square plane fitting those points. The best-fit plane for each slab has been computed too. The attitude of each slab plane with respect to both the adjacent ones and the apron reference plane has been determined by the normal vectors to the surfaces. Faulting has been evaluated as the difference in elevation between the slab planes along chosen sections. For a more accurate evaluation of the faulting value, we have then considered a few strips of data covering rectangular areas of different sizes across the joints. The accuracy of the estimated quantities has been computed too.

## 1. Introduction

Monitoring action of civil infrastructures is one of the significant and complex engineering activities nowadays, due to the need for a continuous evaluation of the state of preservation during their lifetime. New infrastructures are rare, and the monitoring of the existing ones is even more difficult in the case of large infrastructures, such as tunnels, roads, airports, etc. In these cases, it is necessary to conduct an ongoing monitoring action to maintain safe conditions and to avoid deformations, instabilities, or collapses. Moreover, the monitoring actions of civil infrastructures are really useful even for the initial construction phases to check on several activities.

In order to assure the effectiveness of the monitoring action, advanced numerical codes are often used. These assist the technicians during complex and extensive works and they are also indispensable in the case of modernization of an old infrastructure when it is being merged with a new one.

According to the previous considerations, as an example, the construction of a new subway in an urban area and the monitoring of the induced effects on the existing buildings was analyzed in [1]. Furthermore, even in the case of the evaluation of road defects, dedicated monitoring methods are required to support high efficiency repairs [2,3].

In these cases, and in many others, the actions of the monitoring systems are extensive, frequent, and in-depth, and they allow us to evaluate the state of the pavement degradations and, eventually, to plan interventions.

Accordingly, the high performance systems, and in particular those using laser technologies, are involved in monitoring surveys on the infrastructure networks [4].

The analyses are necessarily extensive, due to the size of the infrastructure; they are frequent in order to evaluate continuously the quality of the structures and to control the damage over time. Finally, the activities must be detailed, to allow technicians and experts to become aware of anything that compromises the safety of the infrastructure.

New technologies that use sensors can be very useful to meet the needs, and they can be repeated with definite frequencies to evaluate the progress of the damages [5]. To obtain these objectives, the best instrumentation should be identified.

In the case of an airport pavement management, as an example, the actions must be related to ensure, at the same time, multiple objectives such as an extensive monitoring of the pavement, an effective data quality, and an optimized management of intervention choices, as described in [6]. The document shows one of the main principles of a Pavement Management System strictly applied to a large infrastructure, such as an airport, that consists in evaluating the quality state of the pavements to plan subsequent actions.

The need to apply such a system is due to the heavy work that characterizes these kinds of infrastructures. In the airport field, several studies and research works for good management of the pavements were carried out [7,8,9]. The pavement is generally stressed in heavily used areas.

In order to evaluate eventual defects of the pavement, especially in these large infrastructures, the literature proposes some experiences characterized by the use of laser systems and/or mathematical codes, to verify some important pavement characteristics such as the flatness of concrete surfaces [10,11], the thickness [12] and the curvatures and the deflections caused by repeated traffic loading [13].

In the last decade, many studies have investigated traditional as well as innovative methods to predict damages in rigid pavement used in airport [14]. They classified the different pavement defects in the following ways:

- Cracking (longitudinal, transversal, crocodile),
- Joint defects,
- Superficial defects,
- Instability of the support plane,
- Other typologies of defects.

One of the more interesting problems is related to “faulting value”, faults characterized by different values of elevation in different pavement slab parts of the aprons. In these heavily used areas, the airplane speed is generally very low, the dispersion of trajectories is limited, and the unevenness of the concrete surfaces could be connected to some types of pavement damages such as longitudinal and transversal cracks [15]. Loads repetition and thermal gradients are usually causes of instability of the slabs and cracking on the border lines. Different problems were identified such as daily cycles of frost and thaw, repetition of loads, inadequate thickness of the slab, loss of support and, finally, defects in the original construction.

In 2005 [16], an interesting study on reliability analysis of cracking and faulting prediction was carried out in the New Mechanistic-Empirical design procedure. The guide used common design parameters for traffic, subgrade, environment, and reliability for all pavement types.

A practical method to consider the uncertainties and variations in design and construction was needed so that a new or rehabilitated pavement could be designed for a desired level of reliability.

More recently, a semi-automated faulting measurement for rigid pavements was proposed by Nazef et al. [17].

One year later, Nazef et al. [18] proposed an alternative evaluation practice for an initial assessment of a newly developed automated faulting measurement method.

Tsai et al. [19,20,21] confirm that, because faulting has traditionally been measured manually using hand-held devices such as the Georgia fault meter [22], alternative methods for effectively and safely collecting faulting data should be developed. They proposed a new method to collect faulting data using the 3D continuous pavement profile data acquired with 3D laser technology, and they assessed its feasibility in field test.

Other researchers studied this topic; for instance, Jung and Zollinger [23] proposed a new laboratory-based Mechanistic-Empirical Model for faulting in jointed concrete pavement.

One year later, an automated faulting method was proposed by Mraz et al. [24]. They changed the traditional approach of Florida Department of Transportation (DOT), which measured faulting with a manual fault meter, because extracting the faulting magnitude from a pavement profile collected with an automated high-speed inertial profiler is surely a more efficient and cost-effective alternative. The method detects joints and calculates faulting from longitudinal profile data.

Nowadays, the technicians can refer to the AASHTO (American Association of State Highway and Transportation Officials) standard practice for evaluating faulting of concrete pavements [25] that describes a test method for evaluating in jointed concrete pavement surfaces based on manual methods and automated methods.

More recently, laser scanner methods were used to compute and to verify the concrete slabs faulting values, and were subsequently compared with traditional methods. The use of laser technology to analyze damages [10,26] and to collect faulting data was presented in several works [21,27,28,29].

The aim of our work is to provide a procedure to detect faulting using a Terrestrial Laser Scanner (TLS) instrument in the place of survey traditional methods.

According to the literature review above, the main objective of the work is to demonstrate the effectiveness of the field survey realized with laser scanner technology, and to analyze the displacement of the slabs in airport pavement areas.

The advantage of the proposed method is to extend the measurements, usually manually done on each joint, to the entire paved surface, doing a celerimetric survey with operators placed outside the apron, increasing their safety. The analyses are carried out with post processing techniques.

This paper attempts to define a computation flow chart for TLS data acquired on an apron, in order to obtain the geometrical quantities useful to give the relative and absolute attitude of the single apron slab and after that the faulting. The associated uncertainty has also been computed in order to evaluate the actual statistical significance. The developed procedure has been applied to a test case.

## 2. Case Study

The test area is an apron of a large international airport in Italy whose rigid pavement is made up of concrete slabs 5 m wide by 5 m long. Slabs are connected one to each other by means of joints without dowel bars. The survey covers an area of about 1000 m^2^.

The repeated stress produced by the wheel track of the landing gear to reach the apron, due to a small difference in height in correspondence to the joint, may contribute to both a non-alignment between slabs and a structural damage to the single slab.

For the apron survey, we used a TLS Riegl VZ400 (RIEGL Laser Measurement Systems GmbH, Horn, Austria). The instrument of the TOF (time of flight) type is characterized by a long range, good accuracy and high-speed acquisition. Its main technical characteristics are shown in Table 1. The actual range of the instrument mainly depends on the angle of incidence (angle between the laser beam and the normal to the surface) in addition to the characteristics of the reflectivity and roughness of the scanned object. The received signal strength decreases with increasing incidence angle and influences the accuracy of the distance measure. Therefore, increasing the incidence angle results in a deteriorated signal-to-noise ratio due to elongated laser footprints on the surface as well in an increasing in the distances between successive footprints on pavement and in the reduction of the density of points per surface element.

**Figure 1:**
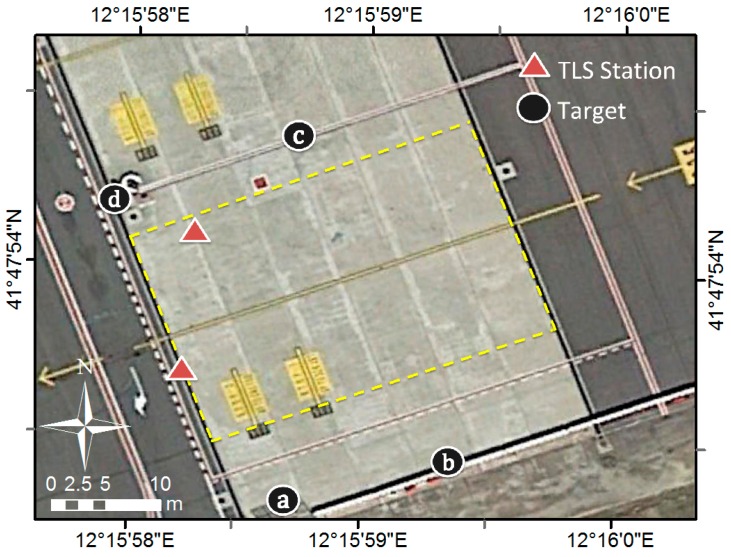
The case study apron (Google Earth image on 5 April 2015).

For the apron survey, the instrument has been set up on a dedicated tripod that is very robust and equipped with a telescopic central pole to allow raising the TLS to a height of 2.20 m, so as to reduce as much as possible the incidence angle value [30]. The apron surface has been measured with the TLS set up on two station points. The two point clouds have been co-registered and georeferenced using four spherical targets with a radius of 0.15 m equipped with a stem of 0.075 m. The position of the station points and the targets are shown in Figure 1.

After the laser scanner survey, we measured the target positions using GNSS (Global Navigation Satellite Systems) receivers in RTK (Real-Time Kinematic) mode. The target barycenter coordinates have been transformed into the reference system used in the airport [31]. The point clouds have been georeferenced to that coordinate system with a six-parameters similarity transformation.

Table 2 reports the summary statistics of the georeferencing residuals obtained using the software package PolyWorks ver. 14 by InnovMetric (Quebec, QC, Canada) [32].

A sub-area made up of 24 slabs have been analyzed (in Figure 1, the area inside the dashed yellow line).

The point clouds belonging to each slab have been cut apart, thus obtaining 24 sub-clouds that describe the surface of each of them. In addition to the single sub-clouds, we processed the whole cloud, made up of all the aforesaid slabs.

**Figure 2:**
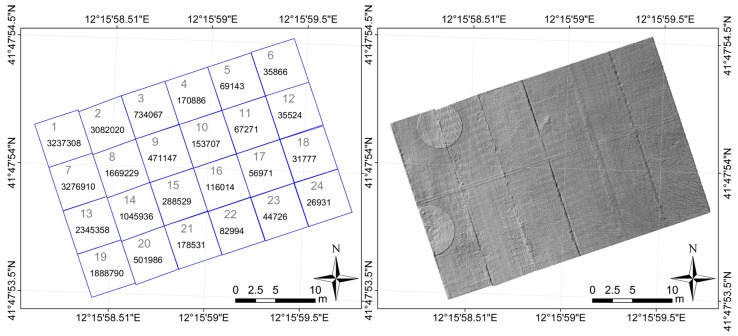
**Left panel**: Partition in slabs of the piece of apron studied along with the numeration used and the number of points belonging to each slab. **Right panel**: DEM displayed in “shadow relief” mode.

The left panel of Figure 2 shows a scheme of the surveyed part of apron and its partition in slabs, along with the numeration used and the number of laser points belonging to each slab. The right panel displays the Digital Elevation Model (DEM) in “shadow relief” mode built starting from the whole point cloud with the software package Surfer ver. 12 by Golden Software, (Golden, CO, USA) [33]. The joints between lines of slabs are clearly visible so as the round shaped areas around the two scan stations, where the point density is smaller than elsewhere.

## 3. Methods

Our elaborations aim to evaluate the faulting value between slabs. Because of this, we have taken into account some aspects of the geometry of the slabs constituting the surveyed apron portion.

In order to compute the faulting, it looks useful to study both the actual relative attitude of the 24 slabs considered and the attitude of the whole apron in order to check the presence of stagnation of water, to verify the comfort and safety condition, and to plan any maintenance activities. Starting from the point clouds, we have fitted 24 plans representative of the single slab surfaces and another one modeling the whole apron.

The process follows several steps:

- Estimates of a plane interpolating the points belonging the individual slabs and their complex;
- Determination of the attitude of the individual slab with reference to the general plan (reference plane);
- Computation of the distance between the points of each slab-plane with reference to the general plane and detection of critical sections;
- Evaluation of faulting on the detected sections;
- Computation of the uncertainty associated with the parameters that define the angular asset.

**Figure 3:**
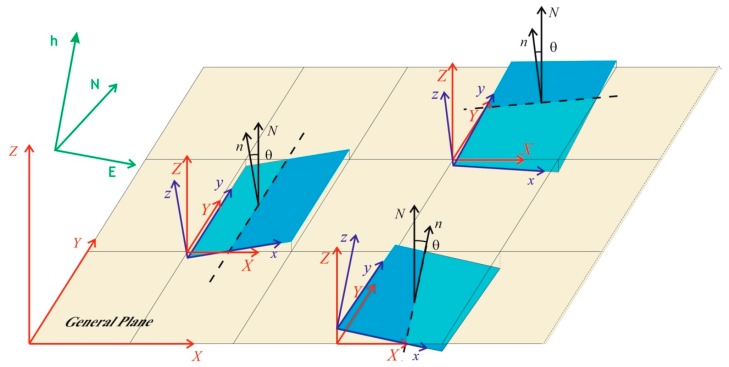
Explanatory drawing of the reference systems used: “absolute” (in green), “general” (in red), “local” (in black) and spatial relationships between them.

A few reference systems have been used, specified below (Figure 3):

- The “absolute” reference system, corresponding with the coordinate reference system (*E*, *N*, *h*), which is used in the airport. In the georeferencing phase, the point clouds acquired on the ground have been framed in this system.
- A “general” Cartesian orthogonal coordinate system (*X*, *Y*, *Z*) with the *X*-, *Y*-axes lying on the “general plane” interpolating the whole point cloud sum of the twenty-four partial clouds, *X*-axis along the direction of the aircraft’s path (solid yellow line in Figure 1), *Z*-axis along the normal to the same plane.
- Twenty-four “local” Cartesian orthogonal systems (*x_i_*, *y_i_*, *z_i_*), each one relative to one single plane-slab, with *x_i_*, *y_i_* axes lying on the plane interpolating the points belonging to the slab, with *z_i_*-axis normal to it.

### 3.1. Plane Fitting and Normal Computation

The portion of survey apron, assumed to be flat, is formed of twenty-four slabs, assumed to be flat too. To compute the attitude parameters of each slab with respect to the general reference plane, we have first computed the equations of both the apron plane and the 24 planes of the single slabs. To this end, the points of the whole cloud have been interpolated to fit a plane, which we will call in the following “general plane”, representative of the geometry of the whole portion of the paved area we studied. The 24 sub-clouds belonging to the individual slabs have been in turn interpolated on planes (24 local “slab-planes”). The attitude of each “local plane” has been studied in relation to the “general plan”.

To estimate the equation of the best-fit plane, we have implemented a fit algorithm based on the Least Squares Estimation (LSE) method. We have run it for each georeferenced point cloud.

The Cartesian equation of the plane:

*h* = *p_1_*∙*E* + *p_2_*∙*N* + *p_3_* (1)

where *E* = East, *N* = North, *h* = ellipsoidal height, *p_i_* are the coefficients of the equation.

This gives us both the estimates of the parameters *p_i_* that define the best-fit plane and their uncertainty. To evaluate the goodness of the fit, we have computed the Root Mean Square Error (RMSE) of the residuals, which indicates the dispersion of the points around the interpolating plane.

The components of the normal to the plane derived from the coefficients of the Cartesian equation of the plane have been obtained using the following relations:

(2) $$n_{E}=p_{1}/M\;,\;n_{N}=p_{2}/M\;,\;n_{h}=-1/M\;;\;M=\sqrt{p_{1}^{2}+p_{2}^{2}+1}$$

In this way, both the value of the components of the normal vector *N* (*N_E_*, *N_N_*, *N_h_*) in the absolute reference system and that for the twenty-four slabs, *n^(i)^*(*n_E_*, *n_N_*, *n_h_*) have been computed.

The attitude of each slab in respect to that of the general plane has been computed considering two parameters:

- The angle ϑ^(i)^ between the normal *n^(i)^* to the plane of the slab and that, *N*, to the reference plane,
- The vector difference between the normal to the *i*-th slab and the “general” normal, ε^(i)^ = *n^(i)^* − *N*, and its projection *ε_π_* on to the general plane.

Considering the scalar product *s* = *n* ∙ *N* = (*n_E_* *N_E_* + *n_N_* *N_N_* + *n_h_* *N_h_*), for the two quantities, we have obtained:

*ϑ* = *arcos*(*n* ∙ *N*) = *arccos*(*n_E_N_E_* + *n_N_N_N_* + *n_h_N_h_*) (3)

*ε_π_* = *n* − (*n* ∙ *N*) *N* (4)

The plane of the single slab is tilted with respect to the general plan along a rotation axis with orthogonal direction *ε_π_*.

### 3.2. Faulting Detection

In order to detect the presence of discontinuity between the slabs, it could be useful in a preliminary phase to effectively locate those areas characterized by a high faulting value, therefore potentially critical. To this end, an algorithm to compute the height difference along the edges has been written and implemented in a code. The output is a text file that contains the cartographic coordinates and the height difference along the edges. If the forenamed value falls in a certain range, the slice of section can be graphically highlighted.

This allows a first rough detection (both graphic and numeric) of the presence of faulting between slabs. Once the critical sections have been located, the faulting value can be computed by sectioning the corresponding portion of point cloud.

#### 3.2.1. Computation of the Difference in Elevation between the Plane-Slab and the General Plane

To compute the difference in elevation along the edge of a slab, the distance between the points of the slab-planes with respect to the general plane has been computed. Once the best-fit plane has been computed, the distance along the normal to the general plane can be computed for each point of the individual local planes.

**Figure 4:**
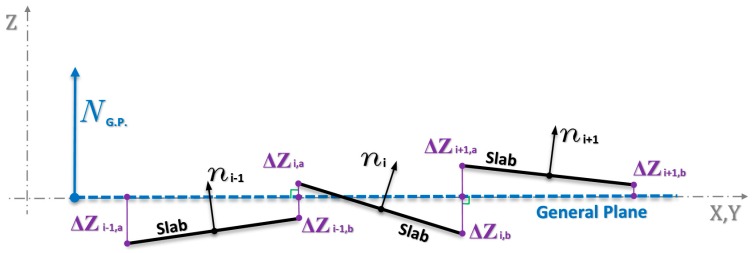
Traces of some slabs and of general plane, normal to the planes, distances ΔZ of the extreme points of the plane-slabs from the general plane (over the joint).

Figure 4 schematically represents the traces of some slabs, their normal lines, the general plane and the distances ΔZ of the extreme points of the adjacent slabs.

The height difference between the points belonging to the plane of the single slab and the general plane gives a picture of the relative assets between the slabs, allowing an immediate visualization of the presence of faulting. Moreover, if a slab sinks, keeping parallel to the original position, a variation of the height Z would arise all the same.

Looking at the display of the distances of the points of the slab, assumed to be a plane, from the general plane, one is able to identify those sections characterized by critical values of discontinuity between adjacent slabs.

#### 3.2.2. Faulting Evaluation along Critical Sections

The method described in Section 3.2.1 aims to speed up the selection of sections to analyze because potentially critical meanwhile has the limit to consider the slab to be a plan, not considering the actual trend of the surface at the edges. The dense survey made with the TLS numerically describes the actual surface of the slab, allowing us to trace an unlimited number of sections and then to compute the faulting on those profiles using a procedure that directly use the point cloud around the detected discontinuity in place of a fit-plane.

Along a number of critical sections, identified using the described method, we have extracted the profiles. One can section the point cloud and extract those ones that fall onto the section line. In this case, the number of points that belongs to the section line obviously depends on the size of the physical area represented by the points, which is in turn determined by the numerical accuracy of the point coordinates.

**Figure 5:**
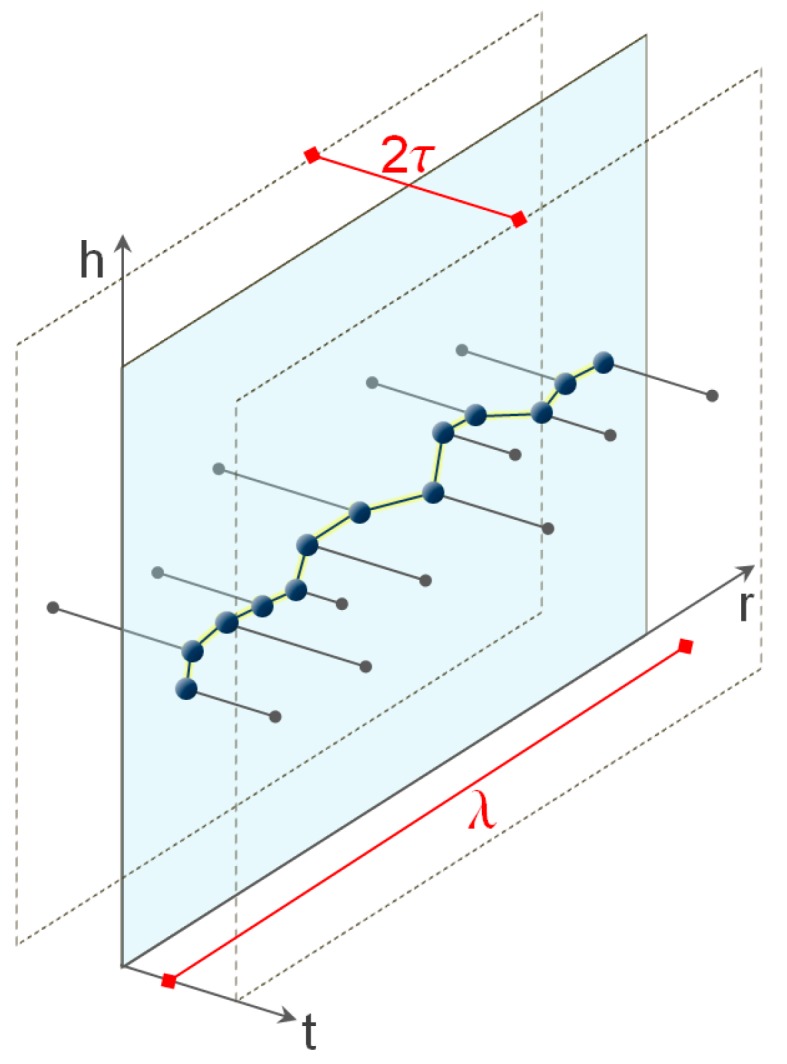
Projection of the points of a strip of point cloud on a vertical plane for a strip of *λ* long and 2*τ* thick.

Otherwise, it is possible to cut a thin portion of data of given thickness across the section and project the points onto a vertical plane passing through the section (Figure 5). We have followed this last procedure.

The variation in thickness of the strip considered leads changes in the number of points that will be part of the cloud. In order to choose the proper thickness of the strip we have computed the number of points that belong to a rectangular area across the joints of side *λ* in the direction perpendicular to the joint and thickness 2*τ* ranging from 20 to 100 mm.

The profiles have been extracted by using a rigid-body transformation. Let us consider a point cloud that has been georeferenced in the absolute reference system, the two points *P_A_* and *P_B_* extreme of the section line A-B where the elevation profile must be extracted, the vertical plane (*r*, *h*) where the section A-B lies.

The code implemented in Matlab ver. 2015a (The MathWorks, Inc., Natick, MA, USA) follows the following steps:

- Translation of axes in the reference coordinate system with origin *A*:
  *x_i_^(A)^* = *P_i_* − *P_A_* (5)
- Computation of the angular distance A → B of the vertical plane:
  (6) $$\theta_{AB}=\arctan\left(\frac{E_{B}-E_{A}}{N_{B}-N_{A}}\right)$$
- Rotation *R_z_*(*ϑ_AB_*) of axes of an angle *ϑ_AB_* around the *Z*-axis:
  *r_i_* = *R_z_*(*ϑ_AB_*) *x_i_^(A)^* (7)
  where *r* = [*r*, *t*, *h*]’.
- Extraction of the set of points *I*, whose coordinate *t* falls within a range *I* of half-amplitude *τ*:
  −*τ* ≤ *t* ≤ +*τ*

Thus, the dispersion of points in the *t*-direction (orthogonal to the plane) has been neglected and therefore we have used all the points belonging to the plane (*r*, *h*). The elevation profile corresponding to section A-B is made up by the piecewise segments joining the points {*r_j_*, *h_j_*; *j* ∈ *I_τ_*}.

### 3.3. Estimating Uncertainty in Parameters

The method we proposed involves the use of quantities derived from measures; therefore, the accuracy of the computed parameters should be verified. The actual significance can be evaluated only analyzing the computed value and the relative uncertainty.

#### 3.3.1. Uncertainties of Attitude Estimates

The use of the Least Squares criterion allows us to compute the standard errors of the estimates of the coefficients of the equation of a plane. In addition, the uncertainty of both the components of the normal to the plane and the quantities derived from them can be obtained by applying the propagation of error formula starting from the co-variance matrix Σ*_p_* of the coefficients a and b.

If *J_np_* is for the Jacobian of the transformation from the coefficients *p* of a plane to the components of the normal and Σ*_n_* for the covariance matrix of the normal to the plane, we have:

(8) $$J_{np}=M^{-3}\;\left[\begin{array}{cc} p_{2}^{2}+1 & -p_{1}p_{2}\\ -p_{1}p_{2} & p_{1}^{2}+1\\ p_{1} & p_{2} \end{array}\right];\;\Sigma_{n}=J_{np}\Sigma_{pp}J_{np}{}^{t}$$

Defining Σ*_N_* as the covariance matrix of the normal *N* to the general plane and assuming that it is not correlated with the normal *n* to the slab-plane, the Standard Deviation (SD) of the scalar product *s* has been obtained through the Jacobian of the transformation from the normals to the scalar products (*J_sn_*):

(9) $$J_{sn}=\left[N_{E}\;N_{N}\;N_{h}\;n_{E}\;n_{N}\;n_{h}\;\right]=\left[\begin{array}{cc} {\underline{N}}^{t} & {\underline{n}}^{t} \end{array}\right]$$

The variance of the angle *ϑ* between the normals can be expressed by the scalar product:

(10) $$\sigma_{\vartheta }^{2}=\sigma_{s}^{2}/(1-s^{2});\;\sigma_{s}^{2}=\left[\begin{array}{cc} {\underline{N}}^{t} & {\underline{n}}^{t} \end{array}\right]\left[\begin{array}{cc} \Sigma_{nn} & 0\\ 0 & \Sigma_{NN} \end{array}\right]\left[\begin{array}{c} \underline{N}\\ \underline{n} \end{array}\right]={\underline{N}}^{t}\;\Sigma_{n}\underline{N}+{\underline{n}}^{t}\;\Sigma_{N}\;\underline{N}$$

The difference *ε* between the two normal, is:

(11) $$\underline{\varepsilon }=\underline{n}-\underline{N}=\left[\begin{array}{cc} I_{3} & -I_{3} \end{array}\right]\left[\begin{array}{c} \underline{n}\\ \underline{N} \end{array}\right];\;\Sigma_{\varepsilon }=\left[\begin{array}{cc} I_{3} & -I_{3} \end{array}\right]\left[\begin{array}{cc} \Sigma_{nn} & 0\\ 0 & \Sigma_{NN} \end{array}\right]\left[\begin{array}{c} I_{3}\\ -I_{3} \end{array}\right]=\Sigma_{nn}+\Sigma_{NN}$$

For its projection *ε_π_* onto the general plane, we have:

(12) $$\begin{array}{l} {\underline{\varepsilon }}_{\pi }=\underline{n}-(\underline{n}\cdot \underline{N})\underline{N}=[I_{3}-(\underline{n}\cdot \underline{N})I_{3}]\left[\begin{array}{c} \underline{n}\\ \underline{N} \end{array}\right]\\ \Sigma_{\varepsilon }=[I_{3}-(\underline{n}\cdot \underline{N})I_{3}]\left[\begin{array}{cc} \Sigma_{n} & 0\\ 0 & \Sigma_{N} \end{array}\right]\left[\begin{array}{c} I_{3}\\ -(\underline{n}\cdot \underline{N})I_{3} \end{array}\right]=\Sigma_{n}+{\left(\underline{n}\cdot \underline{N}\right)}^{2}\Sigma_{N} \end{array}$$

The knowledge of the uncertainty associated with these quantities allows us to estimate the actual significance of the differences between the computed values. If the hypothesis stating that the expected values of the quantities under test are equal is satisfied, the differences between them are zero.

#### 3.3.2. Uncertainty and Significance in Faulting Estimation

The faulting values (*f*) have been computed from the profile data as height differences between the final parts of the straight lines that interpolate the data in correspondence of the joints between the adjacent slabs:

*f* = *Z*_2_ − *Z*_1_ (13)

The uncertainty associated with the computed height derives from the RMSE *σ_o_* associated with the linear best-fitting of data belonging to an area of side *λ* in the direction of the aircraft path over the joints: *σ_Z_* = *σ_o_*.

Afterwards, the uncertainty associated with faulting (*σ_f_*) has been computed via propagation of error formulas starting from the RMSE associated with the heights:

*σ_f_*^2^ = *σ*_*Z*1_^2^ + *σ*_*Z*2_^2^ (14)

The computed faulting value may actually be representative of a step between two adjacent slabs or derived from the accidental errors in the measurements.

We have run a test, based on the null hypothesis *H_o_* of equivalence of heights of the two adjacent slabs; the statistical variable to test is the faulting, *f* = *Z*_2_ − *Z*_1_.

If E{${}_{⋆}$} is the expected value, the null hypothesis that is assumed to be true until proven wrong, is: *H_o_*: *E*{*f*} = 0.

We assume that the actually measured value *f* belongs to a normal distribution with a mean of zero (under the assumption the null hypothesis is true) and the variance is equal to the value *σ_f_*^2^, that is: $f∼N(0,\sigma_{f}^{2})$.

The standardized variable *w_f_* = *f*/*σ_f_* in turn belongs to a standardized normal distribution.

Once the level of significance *α* of the test has been fixed (in the following we assume *α* = 0.1), the region of acceptance will be defined. If the null hypothesis is true, the value of the variable will fall within the confidence interval: −*w_α_*; +*w_α_* corresponding to a (1 − *α*) probability.

Conversely, if the measure’s absolute value |*f*| > *w*_α_, then H_o_ is rejected and the observed height difference can be considered significant. Having assumed *α* = 0.1, the critical value that define the rejection region is *w_α_* = 1.65.

## 4. Results

### 4.1. Attitude of the Slabs

The whole georeferenced cloud has been cut into partial clouds corresponding to the single slabs (see Section 3.1) and the points belonging to each cloud have been used to interpolate a plane. The planar fit has been carried out for the 24 clouds to obtain the equations of the single local plane and, in addition, also for the overall cloud, obtaining the equation of the so-called “general plane”.

It is necessary to assess the accuracy achieved both in the point cloud interpolation on a plane and in the evaluation of the parameters defining the slab attitude, by using the test case’s data.

#### 4.1.1. Accuracy of Planar Fitting

The dispersion of the points around the estimated surface, and, therefore, the effectiveness of the fitting is described by the parameter *s_o_*.

**Figure 6:**
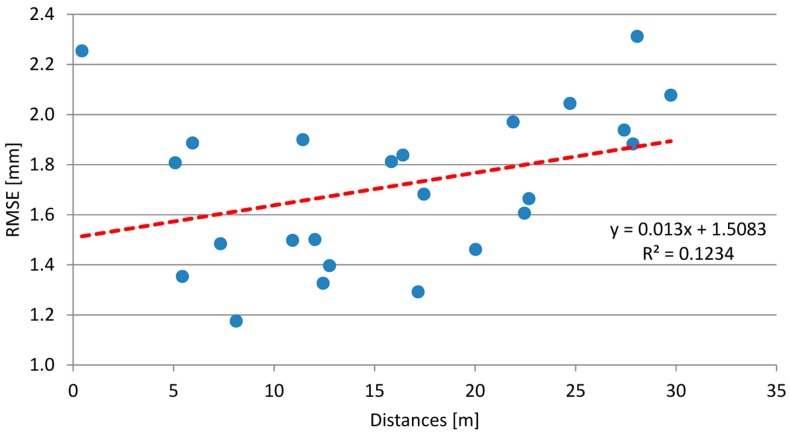
Error in the estimation of the plane that fits the points belonging to each slab, depending on the distance between the centroid and the TLS station.

The farthest slabs from the station points are hit by the laser beams with an inclination angle lower than the nearest ones [34,35]. Consequently, the points considered for the estimation of the coefficients for the farthest slabs are fewer. This affects the fitting quality of the surface and the RMSE ranges from 1 mm to 3.5 mm. Practically, it is a linear function of the distance, as shown in Figure 6.

Subsequently, the attitude of the single slab has been computed with respect to the general plane (2), (3). The geometrical compatibility of a slab with those adjacent is more significant than its attitude with respect to the absolute system, considering also that the whole apron should have a certain slope to allow for the water discharge.

The equations of the lines perpendicular to the local slab-planes have been computed. Table 3 reports the values of the angle *ϑ* between each perpendicular line and the perpendicular to the general plane and the associated SD (9).

Note that the uncertainty is always much lower than the value of the angle, which in some cases exceeds 0.1°, while the SD does not reach 0.002° even for the farthest slabs.

Previous results allow us to design the survey in order to increase the effective distance between the slabs and the TLS station, maintaining the accuracy of the interpolation.

#### 4.1.2. Accuracy in Estimating the Parameters of Attitude of the Slabs

The magnitude and direction of the vector *ε* (difference between the two perpendicular lines) gives the attitude of each slab relative to the general plane. The axis of rotation of the slab is perpendicular to the projection of the vector *ε* onto the general plane.

**Figure 7:**
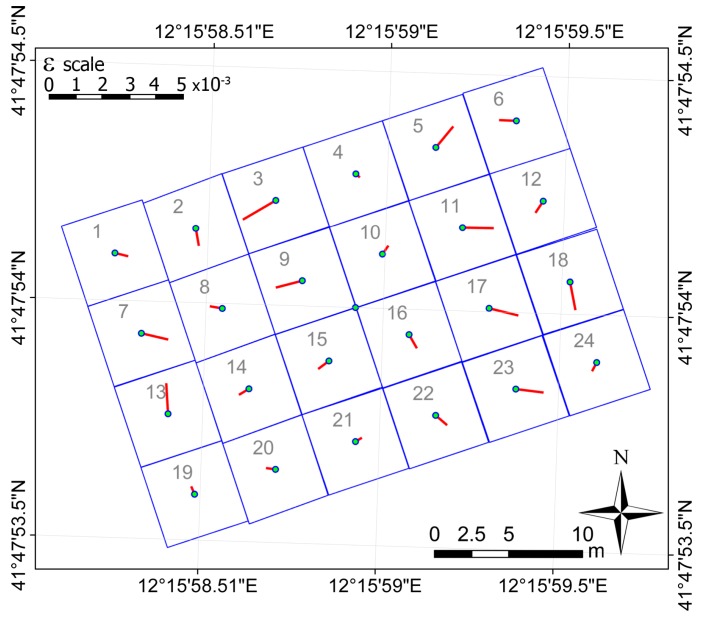
Magnitude and directions of the projection of the vector *ε* differences between the perpendiculars to the single slab and the general plane onto the general plane.

The components of the orthogonal projection of the vector *ε* onto the general plane are summarized in Table 4, together with the ratio between their value and the relative SD, computed according to the Equation (11). The uncertainty is about 200 times smaller than the value of the component in this case too. Figure 7 shows the projection of the vector onto the general plane; the length of the line is proportional to the angle of inclination.

### 4.2. Faulting Detection

#### 4.2.1. Faulting Evaluation Using Planar Fit

**Figure 8:**
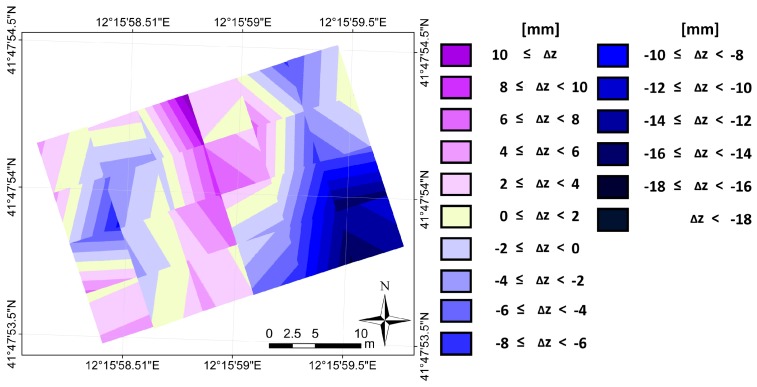
Faulting’s graphical display.

The difference in height between the points belonging to the plane fitting the single slab and the corresponding points of general plane issues an overview of the faulting between slabs. The height differences at the joints corresponds to the faulting. Faulting value is graphically displayed in Figure 8; the chromatic scale highlights the faulting values at the joints, by steps of 2 mm, allowing an effective visualization of the presence of faulting.

**Figure 9:**
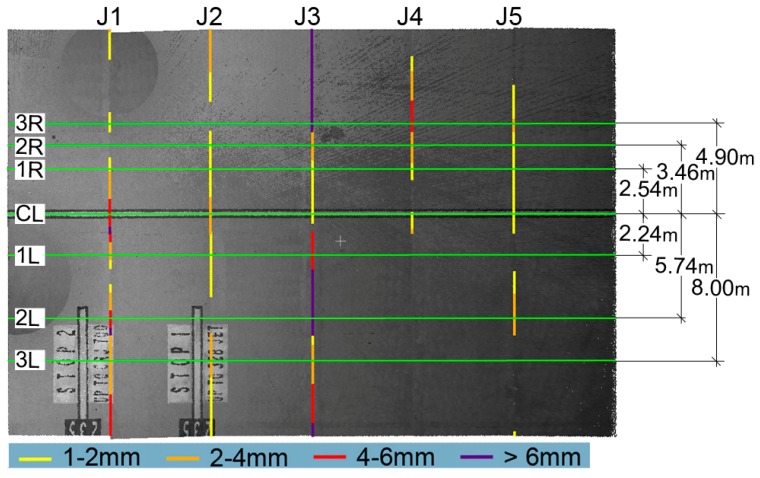
Left panel: graphical display of the linear sections of the joints perpendicular to the airplane path where the faulting values fall between certain values (shown in the legend).

In Figure 9, the green lines highlight a few sections significant for the evaluation of the faulting; in addition to the section over the center line (CL), three sections have been traced on its right (1R, 2R, 3R) and three on its left (1L, 2L, 3L). The colored lines graphically displayed a certain amount of faulting evaluated in correspondence of the joints. Along these section lines, we have extracted the profiles. The faulting values computed in correspondence of the joints (from J1 to J5) are reported in Table 5. The faulting values computed in this way are not fully representative of the edge characteristics since the slab has been considered as a whole, which is a plane.

#### 4.2.2. Faulting Evaluation Using Points Closeness to the Edges of the Slabs

Otherwise, the faulting evaluation may derive from the difference in height between two portions of adjacent slabs close to the joint. The heights of the edges of each slab have been computed according to the procedures described in Section 3.2.2, by linear interpolation of a short section of edge.

**Figure 10:**
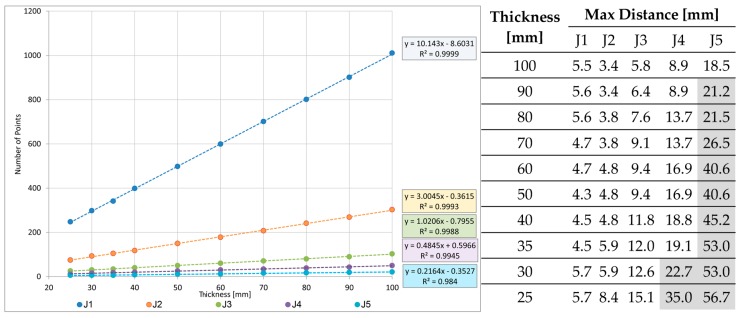
Relationship between the points contained in a 150 mm long rectangular area over the joints and the thickness of the area. Left panel: point density. Right panel: maximum distance between couple of points. Values exceeding 20 mm are highlighted in grey.

The left panel of Figure 10 shows the graph of the point density in a rectangular area over the joints 150 mm long and from 20 to 100 mm wide, computed in correspondence of the center line. Density increases quite linearly. Over the farthest joint J5 from the laser stations, the maximum value of density is 0.07 points/cm^2^, over the 150 × 100 mm area.

We also have computed the maximum distance between couple of points, for a number of strip thicknesses (right panel of Figure 10). For J5, the inter-distance results 40 mm for a 50 mm thickness, whereas it decreases to less than 20 mm for a 100 mm thickness. For J4, to reach an inter-distance lesser than 20 mm, the strip thickness should be 30 mm at least. Considering these results and taking into account the average dimensions of the footprint area of an aircraft front wheel, we have decided to choose a thickness of 100 mm in order to assure a sufficient density of points even for the farther joints.

**Figure 11:**
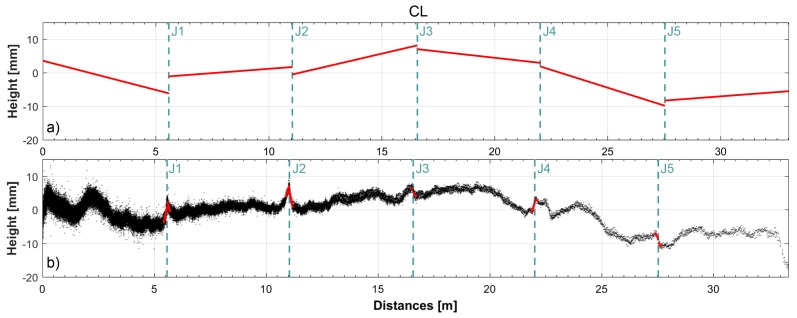
Profiles extracted along the center line. (**a**) profile extracted considering flat the slab surface. (**b**) point cloud (in black) and its linear interpolation for a 150 mm segment as of the joint (in red).

Figure 11 displays the profiles extracted along the center line. In the top panel, the profile extracted is shown considering the slab surface flat, whereas, in the bottom panel, the point cloud and its linear interpolation for a 150 mm segment as of the joint (red segments) is shown.

The differences between the results of the two different evaluations derives from the difference between data processing: in case a planar fit is used to interpolate all the points of each slab, whereas, in the other case, a linear fit is used to interpolate a profile extracted using only a small area adjacent the joint.

It could be interesting to analyze the trend of the faulting near the joints from the numerical point of view. Table 6 reports the values of faulting *f*, computed on the point cloud in a 100 × 150 mm area over each joint, the associated SD *σ_f_* and their ratio *w_f_* = *f*/*σ_f_*. The computed faulting value varies from 0.1 to 5.1 mm and the associated SD varies from 0.7 to 2.2 mm.

In Section 3.3.2, we have established that the faulting value is esteemed significant at a level of confidence of 90% if the ratio *w_f_* exceeds 1.65. According to this assumption, nine faulting values are significant (26%); for these, it makes sense to verify if the values exceed the critical threshold. In all other cases, the measured values are not significantly different from zero, at the confidence value of 90%.

Obviously, the choice of a lower level of confidence, for example of 80%, defines the critic value of the ratio *w_f_* = 1.28 and increases up to 14 the number of faulting values that have been established as significant.

## 5. Discussion

The use of georeferenced points derived from a laser scanner survey of the slabs part of the apron considered in this study allows for defining effectively a planar model of the slab. The goodness of fit is assessed by the RMSE value, in the order of magnitude of a few millimeters and depending on the distance between the scan station and the points on the apron surface.

The knowledge of the trend, which is almost linear, can be useful when designing the survey, to choose the limit distance, beyond which a linear fit is no longer appropriate.

A key factor to consider when designing the surveys is the height of the instrument with respect to the ground, which must be raised using an elevating tripod. The higher the TLS, the greater the height than the maximum effective range, the minimum angle of incidence being equal.

Thanks to the large number of laser points available, the accuracy of the computation of the normal lines to the planes is very high. It means that both the computation of the angle between the perpendicular to each plane and the one to the general plane and of the vector “difference” are affected by very small errors and their values can be considered fully significant.

The accurate knowledge of the relative attitude of the apron slabs to each other allows for effectively studying the height changes of the various points of the plane-slab and then the prospective presence of faulting in the directions of major interest for the aircraft path. The developed software allows for evaluating the faulting values along a number of selected profiles, allowing the maintainer to locate the critical ones.

It is evident that the use of a planar surface to model the slab does not take into account the trend of the effective surface surveyed with a high level of accuracy; looking at Figure 11, the difference between the effective profile of the surface and the interpolating plane used is evident, even if the differences are on the order of the millimeters.

It is therefore more significant to evaluate the faulting at the edges of the slabs, using only the points acquired on a small final strip where the step between adjacent slabs is felt by the aircraft wheels, rather than all data.

The faulting values derived from surveying operations are usually very small, and then the uncertainties related with both measurements and computation must be computed in order to evaluate if the observed value derives only or mainly from measurement errors, or it represents a significant signal.

First, we adopted the above-mentioned procedure to strips 300 mm long (150 + 150 mm over the joint); the results are shown in Table 6. The comparison between the faulting values computed using the whole local slab-planes (right panel of Figure 10) and those computed using the 300 mm strips (Table 6) is provided in Table 7. The values of the height difference computed using the strips sometimes are decreased: they are lower than 1 mm in the 43% of the measures while they exceed 2 mm only in the 17% of the cases (highlighted in grey in Table 7).

Over the joint J3, section 2L and over the joint J4, section 3R, the differences between faulting values slightly exceed 5 mm. The warning of high values of faulting has subsided for the joints J1-CL, J4-3R, J4-2R and J5-2L, whereas it has been confirmed for the joints J3-3R and J3-2L.

The analysis made considering only the edges of the slab is more suitable since it takes into account the deformations that occur and gives the effective value of the step height.

Since the number of points present in the considered strip depends on its size, we have studied the behavior of a slightly longer strip, 200 + 200 mm over the joint, with the aim of evaluating how much the faulting value varies with the size of the point cloud to interpolate. The faulting values have been computed running the procedure on a 200 × 100 mm dimension area for any edge-slab. Table 8 reports the computation results with the associate SD.

In this case, the most part of faulting values is slightly higher even though the associated standard deviation values are almost the same. Thirteen faulting values are now statistically significant (*w_f_* > 1.65), more numerous than in the previous case (green cells in Table 8).

**Figure 12:**
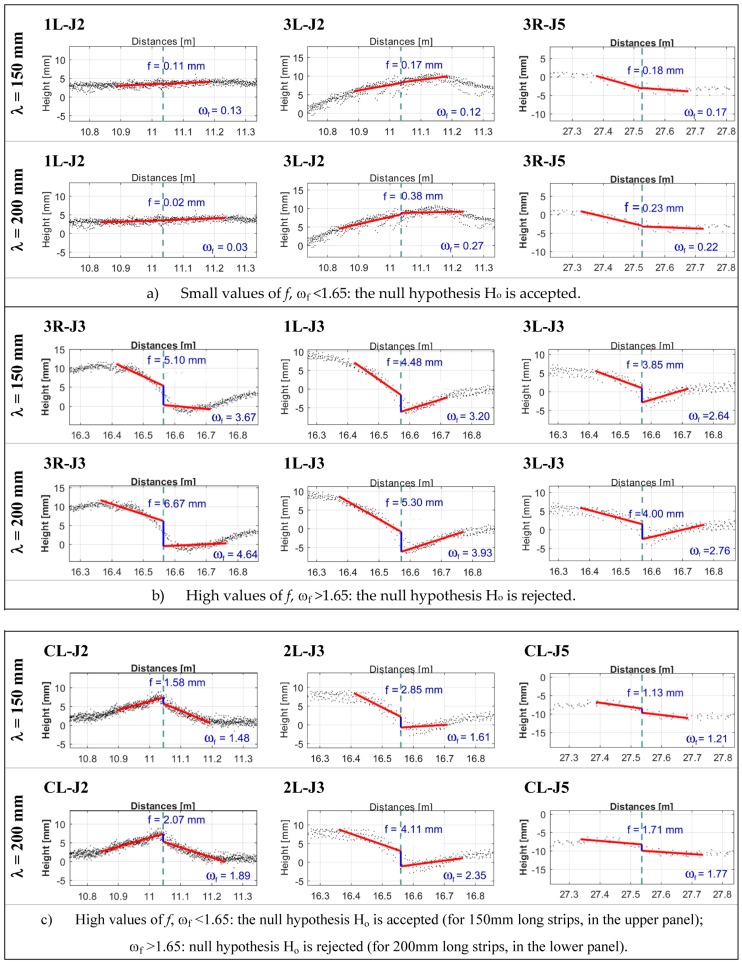
Display of the faulting values computed using the segments interpolating the point clouds for strips of 150 + 150 mm (upper panels) or 200 + 200 mm (lower panels) over the joints.

In Figure 12a–c, for a number of joints, is shown a comparative display of the faulting values computed using the segments interpolating the point clouds for strips of 150 + 150 mm (upper panels) or 200 + 200 mm (lower panels) over the joints. In the first three cases (Figure 12a), the value of *f* is so small that the null hypothesis H_o_ (no step between the slabs) is fully accepted in both cases.

For other joints, the value of *f* (Figure 12b) is high, and, even in this case, the decision is the same in both cases: hypothesis H_o_ must be rejected. It follows that there is a step between the slabs. Conversely, for intermediate values of *f*, the choice of the size of the strip being analyzed is not irrelevant. Indeed, in the four cases displayed in Figure 12c, the computed faulting value is not considered statistically significant if the shorter strip is used, whereas it is in the case of the longer strip choice.

Most of the time, the use of *λ* = 200 mm leads to an increase in the number of cases where the measured value is significantly different from zero and therefore indicative of a step actually present among the slabs along the section considered. Obviously, it should be underlined that, although significant, the evaluated value for the step height can be so small that it does not give rise to any alarm and consequent action. The method we proposed selects those measured values that could be reasonably compared with the alarm threshold.

## 6. Conclusions

In this note, we proposed a data processing method for the evaluation of both the relative attitude of adjacent slabs of an apron and the faulting between them using as input data the point cloud surveyed by means of a widespread instrument, the TLS.

Our method is based on the cut of the georeferenced cloud into sub-clouds representative of the single slabs and on the writing of the equation of the planes that interpolate the clouds both for the apron (general plane) and for each slab (local planes). We also give the formulas for computing the uncertainty associated both with the normal vectors of the best-fitting planes and with the angle between the normals (to the general plane and the local ones).

The accuracy related to the mentioned quantities must be evaluated in order to determine the statistical significance of the estimated values.

For our test case, the value of the angle between the normal to any plane-slab and the normal to the general plane that is assumed as reference is much greater than the uncertainty associated to both the raw measures and the derived quantities, also thanks to the high number of points in the cloud.

The computation and the display of the height difference between each slab and the reference apron, both modeled using a planar surface, has resulted in being useful to the knowledge of the relative attitude between slabs and therefore allows us to narrow the number of profiles along which compute the faulting.

In order to compute the faulting value, we suggested to use small portions of data belonging to two adjacent areas over the joint rather than the entire slab-plane. It is not trivial to define the size of the rectangular strip of data to use first for the projection of them onto a vertical section plane and then for the linear best fit. Our tests made on an apron of an international airport led us to decide to choose a strip 100 mm thick and 200 mm long rather than 150 mm long.

The TLS survey of the apron is just like a “picture” of the apron taken at a certain epoch that can be stored in a database and reused later for monitoring purposes in order to rebuild the “history” of a failure. The acquired data, once they have been co-registered and georeferenced and taking care to provide metadata containing information about the acquisition process, are able to document the maintenance status of the apron at that moment.

The proposed method makes use of celerimetric measurements in place of hand-held devices, keeping the accuracy level safe on their own. This allows for making available a large-scale observation of the infrastructure behavior and therefore the possibility of using pre-existing surveys to reconstruct the pavement degradation and its punctual evolution over time.

We proposed a new procedure aimed at defining monitoring actions fully suitable for systematic use by the maintenance authority. In order to optimize the procedure, the activities we want to develop in the future are:

- Comparison between both hand-held devices and LiDAR mobile systems;
- Improvement of the operating procedure for field survey. In better detail, the TLS should be set up on a dedicated tripod equipped with a telescopic central pole to allow raising it to a large height. Moreover, the actual instrument range must be chosen taking into account that the RMSE of the plane fitting increases as the distance increases (see Figure 6);
- Software engineering to make semi-automatic the detection of the directions where faulting likely happens (see Figure 8);
- Building a database for the management of data coming from surveys repeated over time.

## Figures and Tables

**Table 1:** Riegl VZ400 technical characteristics.

Laser System	Riegl VZ400
Beam Divergence	0.35 mrad
Beam Diameter	35 mm @ 100 m
Range	1.5 up to 600 m (long range mode)
Angle Measurement Resolution	0.0005°
Vertical Field of View	100°
Horizontal Field of View	360°
Vertical Resolution	0.0024°–0.288°
Horizontal Resolution	0.0024°–0.5°
Accuracy	0.005 m
Precision	0.003 m
Scanning Speed	300 Hz up to 122,000 points/s

**Table 2:** Summary statistics of the georeferencing residuals.

Summary Statistics	Values (mm)
Max. Error	11
Min. Error	2
Mean Error	6
Standard Deviation	4

**Table 3:** Values of the angle *ϑ* between the local orthogonal lines and the perpendicular to the general plane together with the associated SD.

Slab	Angle (Deg)	SD (Deg)	Slab	Angle (Deg)	SD (Deg)
1	0.05	0.0001	13	0.11	0.0001
2	0.06	0.0001	14	0.05	0.0001
3	0.14	0.0002	15	0.05	0.0002
4	0.02	0.0004	16	0.06	0.0005
5	0.10	0.0008	17	0.11	0.0008
6	0.07	0.0013	18	0.11	0.0014
7	0.10	0.0001	19	0.03	0.0001
8	0.05	0.0001	20	0.04	0.0001
9	0.10	0.0002	21	0.02	0.0003
10	0.04	0.0005	22	0.06	0.0005
11	0.11	0.0008	23	0.10	0.0011
12	0.05	0.0012	24	0.04	0.0015

**Table 4:** Left column: summary statistics of the component values of the orthogonal projection of the vector ε onto the general plane and of the ratio between the components and their SD (right column).

	Component Values			Ratio between Components and SD		
	|X|	|Y|	|Z|	X	Y	Z
max	0.00217	0.00189	0.000017	976	1206	1075
min	0.00008	0.00001	0.000001	12	3	12
mean	0.000898	0.000604	7.13 × 10^−6^	230	189	240

**Table 5:** Faulting values evaluated in correspondence of the joints.

	Faulting Values Using Planar Fit (mm)				
	J1	J2	J3	J4	J5
3R	1.4	0.2	8.8	5.5	2.1
2R	0.5	1.3	2.1	2.7	1.2
1R	1.8	1.6	1.8	1.6	1.3
CL	5.1	2.2	1.1	1.1	1.6
1L	2.4	1.2	5.4	0.3	0.2
2L	4.9	0.9	7.9	0.4	3.1
3L	3.5	2.1	2.8	0.5	0.3

**Table 6:** Faulting values, associated SD and ratio between faulting and SD computed taking into account the point cloud belonging to a 150 × 100 mm area. *w_i_* values exceeding 1.65 are highlighted in grey.

	J1			J2			J3			J4			J5		
	*f* mm	*σ_f_* mm	*w_f_*	*f* mm	*σ_f_* mm	*w_f_*	*f* mm	*σ_f_* mm	*w_f_*	*f* mm	*σ_f_* mm	*w_f_*	*f* mm	*σ_f_* mm	*w_f_*
3R	2.1	2.2	0.94	1.2	1.1	1.08	5.1	1.4	3.67	0.3	1.0	0.31	0.2	1.1	0.17
2R	2.1	1.3	1.64	2.2	1.0	2.35	1.7	0.8	2.13	0.2	0.9	0.21	0.4	1.0	0.39
1R	0.8	1.1	0.75	0.3	1.1	0.24	1.8	0.9	2.07	1.2	0.8	1.52	0.3	0.7	0.38
CL	1.5	1.5	1.00	1.6	1.1	1.48	1.2	1.0	1.15	1.00	0.9	1.13	1.1	0.9	1.21
1L	0.7	1.1	0.61	0.1	0.8	0.13	4.5	1.4	3.20	0.6	0.8	0.83	1.8	0.8	2.11
2L	3.3	1.3	2.62	2.5	1.1	2.31	2.9	1.8	1.61	0.4	1.2	0.35	0.6	1.2	0.54
3L	2.0	1.5	1.31	0.2	1.4	0.12	3.9	1.5	2.64	0.3	1.4	0.18	2.1	1.8	1.15

**Table 7:** Differences of faulting values obtained using planar model or 300 mm long strips. Faulting values exceeding 2 mm are highlighted in grey.

Section	Differences of Faulting Values (mm)				
	J1	J2	J3	J4	J5
3R	−0.7	−1	3.7	5.2	1.9
2R	−1.6	−0.9	0.4	2.5	0.8
1R	1	1.3	0	0.4	1
CL	3.6	0.6	−0.1	0.1	0.5
1L	1.7	1.1	0.9	−0.3	−1.6
2L	1.6	−1.6	5	0	2.5
3L	1.5	1.9	−1.1	0.2	−1.8

**Table 8:** Faulting, RMSE and ratio fault/*σ*Δ_sx,dx_ computed using the segment, which linearly interpolate the point cloud in an area of 200 × 100 mm. *w_i_* values exceeding 1.65 are highlighted in grey.

	J1			J2			J3			J4			J5		
	*f* mm	*σ_f_* mm	w_f_	*f* mm	*σ_f_* mm	w_f_	*f* mm	*σ_f_* mm	w_f_	*f* mm	*σ_f_* mm	w_f_	*f* mm	*σ_f_* mm	w_f_
3R	2.4	2.1	1.11	1.6	1.1	1.46	6.7	1.4	4.64	0.7	1.0	0.71	0.2	1.1	0.22
2R	2.8	1.4	2.08	2.5	1.0	2.57	2.7	0.9	2.88	0.3	0.9	0.28	1.0	1.0	1.01
1R	0.7	1.2	0.69	0.3	1.0	0.29	1.9	0.8	2.27	1.3	0.9	1.52	0.5	0.8	0.61
CL	1.6	1.5	1.11	2.1	1.1	1.89	1.4	1.0	1.38	1.4	0.9	1.59	1.7	1.0	1.77
1L	0.8	1.1	0.78	0.02	0.8	0.03	5.3	1.4	3.93	0.5	0.8	0.65	2.5	1.0	2.69
2L	4.1	1.3	3.15	2.8	1.1	2.58	4.1	1.8	2.35	0.7	1.1	0.64	1.2	1.2	1.03
3L	1.8	1.5	1.18	0.4	1.4	0.27	4.0	1.5	2.76	0.4	1.5	0.29	2.0	1.7	1.20
